# Daratumumab as rescue therapy in refractory recurrent FSGS after kidney transplantation: a case series

**DOI:** 10.3389/ti.2026.16693

**Published:** 2026-06-10

**Authors:** Parthenopi Despina Avaniadi, Aylin Akifova, Eva Vanessa Schrezenmeier, Sophie Lüdemann, Georgios Eleftheriadis, Ayse Selin Güneytepe, Linde Gao, Friederike Bachmann, Felicitas E. Hengel, Anne Mühlig, Nicola M. Tomas, Tobias B. Huber, Kai-Uwe Eckardt, Klemens Budde, Fabian Halleck

**Affiliations:** 1 Department of Nephrology and Medical Intensive Care, Charité – Universitätsmedizin Berlin, Berlin, Germany; 2 III Department of Medicine, University Medical Center Hamburg-Eppendorf, Hamburg, Germany

**Keywords:** anti-CD38-antibodies, daratumumab, focal segmental glomerulosclerosis, kidney transplantation, recurrent disease

Dear Editors,

Recurrent focal segmental glomerulosclerosis (FSGS) remains one of the most challenging complications after kidney transplantation, frequently leading to early graft dysfunction and premature graft loss [1–3]. Despite advances in immunosuppressive therapy, treatment options remain limited, particularly in patients refractory to standard therapies including plasmapheresis and B-cell–depleting strategies. In this context, plasma cell–targeted therapies such as daratumumab have emerged as a promising strategy in refractory cases [4–7]. However, current evidence is limited to small case series and heterogeneous cohorts, and the effectiveness of daratumumab across different clinical scenarios remains unclear.

We report a single-center case series of six kidney transplant recipients with biopsy-proven recurrent FSGS who were refractory to conventional treatment and received daratumumab as rescue therapy. All patients had kidney failure due to primary FSGS with biopsy-confirmed recurrence after transplantation. Electron microscopy demonstrated a podocytopathy with diffuse podocyte foot process effacement in all cases. Genetic testing using a targeted NGS panel was performed in four patients and did not reveal a genetic cause of FSGS.

All patients received induction therapy with basiliximab followed by standard triple maintenance immunosuppression consisting of tacrolimus, mycophenolate mofetil, and corticosteroids. All patients were dialysis-dependent and anuric prior to transplantation, no pre-emptive transplantations were performed; one patient had previously undergone kidney transplantation. Following biopsy-confirmed recurrence of FSGS, five patients were switched from tacrolimus to cyclosporine. All patients received anti-proteinuric therapy with angiotensin-converting enzyme inhibitors or angiotensin receptor blockers at maximally tolerated doses. One patient additionally received an SGLT2-inhibitor.

Prior to daratumumab, all patients were treated with plasmapheresis and anti-CD20 therapy (rituximab in all cases with escalation to obinutuzumab in one patient), reflecting current standard approaches, but failed to achieve sustained remission. Treatment timing varied, reflecting real-world practice. The interval between initiation of anti-CD20 therapy and the first daratumumab administration was a median of 110 days (IQR 72–133).

Daratumumab was administered subcutaneously at a fixed dose of 1800 mg, with patients receiving between three and six administrations. Median follow-up after the last daratumumab administration was 12.3 months (IQR 11–14.7).

Following daratumumab treatment, four patients achieved complete remission and two achieved partial remission, accompanied by stabilization or improvement of graft function. These responses were observed despite prior failure of multimodal therapy and may reflect meaningful treatment effects in a highly refractory population, likely driven by a synergistic effect of B-cell and plasma cell depletion. One patient experienced a relapse 12 months after treatment but regained complete remission after a single additional dose of daratumumab, suggesting that retreatment may be effective in selected cases. Infectious complications, including cytomegalovirus infection, hepatitis E virus infection, herpes zoster, and bacterial infections, occurred in three patients. All infections were mild to moderate, managed as outpatients, and resolved without sequelae, indicating an acceptable safety profile despite intensive immunosuppression.

Importantly, our cohort reflects a broad spectrum of clinical presentations and disease trajectories. Recurrence occurred within days of transplantation in most patients, consistent with previous reports, but included one patient with delayed clinical progression requiring treatment escalation more than 4 years post-transplant. Notably, this patient had an early histological recurrence but remained clinically stable 4 years before developing overt nephrotic syndrome with graft dysfunction. This highlights the dynamic nature of recurrent FSGS and suggests that disease activity may evolve before manifesting clinically. The longitudinal course of proteinuria and graft function following daratumumab therapy is illustrated in Figure 1.

**FIGURE 1:**
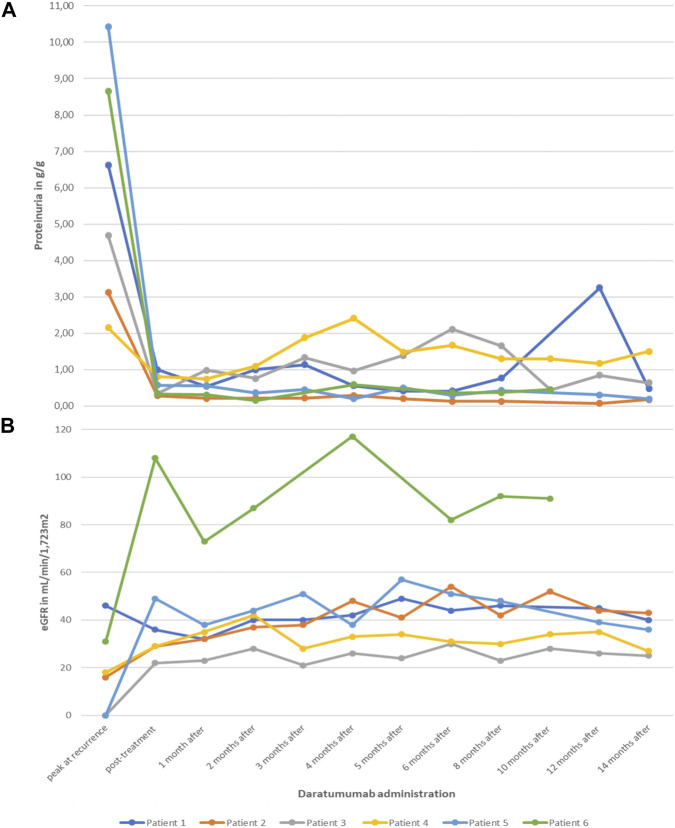
Longitudinal course of proteinuria and graft function during and after multimodal therapy for recurrent post-transplant FSGS. Each line represents an individual patient with recurrent post-transplant FSGS (n = 6). **(A)** Urine protein-to-creatinine ratio (UPCR; g/g) values are shown at peak proteinuria at recurrence, after completion of treatment, and during follow-up. Peak values were obtained prior to daratumumab initiation, although some patients were already receiving plasmapheresis or rituximab, with escalation to obinutuzumab in one patient. Post-treatment values were assessed at a median of 16 days (IQR 7–18) after the last daratumumab administration. Four patients achieved complete remission (<0.5 g/g creatinine), and two achieved partial remission (<2 g/g creatinine with ≥50% reduction in proteinuria). Patient 1 experienced a relapse 12 months after the last daratumumab dose and received a single additional administration. **(B)** Estimated glomerular filtration rate (eGFR; mL/min/1.73 m^2^) values are shown at recurrence, after completion of treatment, and during follow-up. eGFR was calculated using the CKD-EPI equation. Treatment consisted of plasmapheresis, rituximab/obinutuzumab, and daratumumab. Follow-up time points at 1, 2, and 3 months and thereafter refer to the interval since the last daratumumab administration.

In contrast to previously reported case series, our observations emphasize that daratumumab may have potential across broader clinical scenarios than previously appreciated. While prior reports have focused on early recurrence or selected refractory cases, our cohort captures both early and delayed treatment settings and diverse disease trajectories. This provides additional insight into the potential role of plasma cell–targeted therapy in clinical practice.

Recent multicenter analyses have reported encouraging remission rates with combined B-cell and plasma cell–targeted strategies [7]. Our findings are consistent with these observations but extend them by providing detailed longitudinal clinical data in a well-characterized cohort, allowing a more granular and clinically relevant assessment of treatment response. In particular, the inclusion of patients with delayed disease progression and prolonged disease courses prior to treatment initiation suggests that the therapeutic window for daratumumab may be broader than previously assumed.

Circulating anti-nephrin autoantibodies have recently been proposed as potential biomarkers and possible mediators of recurrent podocytopathies [8, 9]. Anti-nephrin autoantibodies were serologically assessed in all patients at recurrence during active nephrotic disease and were negative in all cases. These findings argue against a dominant role of anti-nephrin–mediated mechanisms in these cases and further underscore the biological heterogeneity of recurrent FSGS. They also suggest that additional, as yet unidentified circulating factors may contribute to disease pathogenesis in a subset of patients. However, additional podocyte-associated markers were not systematically assessed and therefore cannot be evaluated in this cohort.

The mechanism of action of daratumumab in this setting likely extends beyond plasma cell depletion. CD38 is expressed on multiple immune cell subsets, including plasma cells, activated B cells, T cells, natural killer cells, and dendritic cells. Targeting CD38 may exert broader immunomodulatory effects influencing circulating permeability factors [10]. While the precise mechanisms remain incompletely understood, the consistent clinical responses observed in our cohort support further investigation of CD38-directed therapies in recurrent FSGS.

Our study has several limitations, including its retrospective design, small sample size, short follow-up, and lack of systematic B-cell and plasma cell count data, precluding conclusions regarding long-term outcomes. In addition, treatment strategies were not standardized but reflected real-world clinical practice. The absence of a control group limits attribution of treatment effects to daratumumab alone. Nevertheless, the consistent responses observed across a heterogeneous patient population provide clinically relevant insights into treatment effectiveness in a salvage setting.

In conclusion, daratumumab may represent a promising therapy in patients with recurrent FSGS after kidney transplantation who are refractory to standard treatment. Our findings suggest potential benefit across diverse clinical scenarios, including delayed treatment escalation and prolonged disease courses, and support further evaluation in prospective, multicenter studies.

Sincerely,

Parthenopi Avaniadi.

## Data Availability

The original contributions presented in the study are included in the article/supplementary material, further inquiries can be directed to the corresponding author.
